# Giant anal warts

**DOI:** 10.1002/ccr3.3021

**Published:** 2020-06-08

**Authors:** Francesk Mulita, Panagiotis Tavlas, Evangelos Iliopoulos, Ioannis Maroulis

**Affiliations:** ^1^ Department of Surgery General University Hospital of Patras Achaia Greece

**Keywords:** anal wart, high‐grade squamous intraepithelial lesion

## Abstract

Anal lesions can occur due to infectious and neoplastic etiology, and a prompt and multidisciplinary approach may prevent poor outcomes.

## Graphical abstract

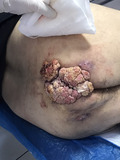

## 1 CASE DESCRIPTION

**FIGURE 1:**
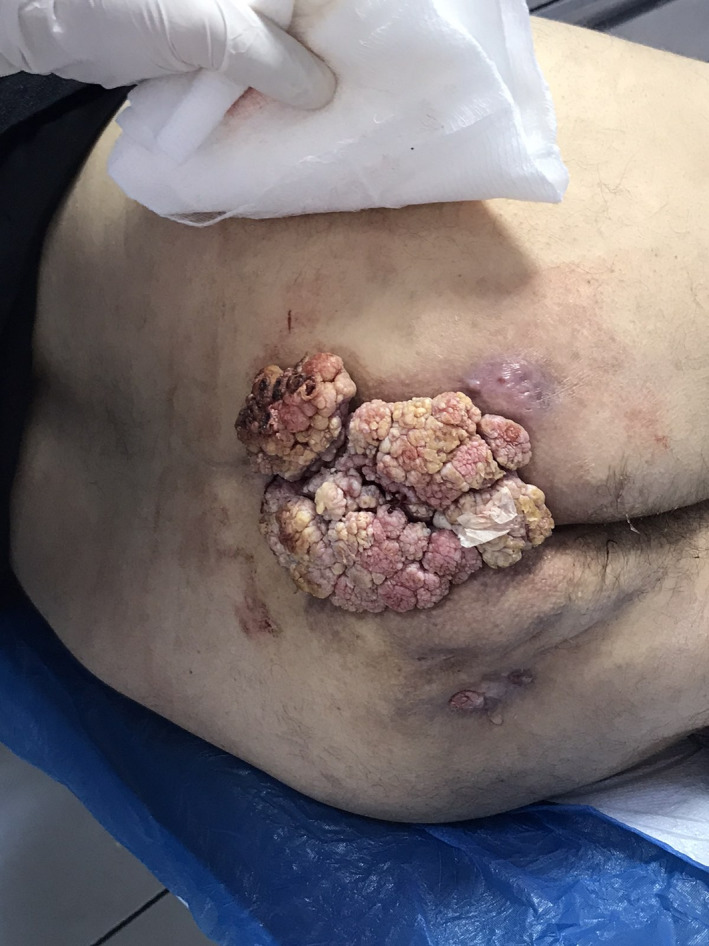
Large anal warts arising from anus

A 70‐year‐old, HIV‐negative male presented with a neglected slow‐growing anal wart for many years with bleeding and pruritus. Examination confirmed a large anal mass with ulceration (Figure 1). Blood analysis revealed hemoglobin of 6.3 g/dL, and colonoscopy was normal.

Computed tomography scan revealed a 14 × 10 × 8 cm mass with no metastasis.

A deep core biopsy of the tumor confirmed a high‐grade squamous intraepithelial lesion (HSIL) and carcinoma in situ with p16 positivity suggestive of high‐risk HPV subtype.

High‐grade squamous intraepithelial lesions (HSILs) are considered premalignant and can progress to anal cancer. The progression risk is elevated in certain high‐risk groups, including patients with infection with high‐risk HPV strains (types 16 and 18). Limited data are available comparing different treatment modalities in men with HSIL. Multidisciplinary approach is necessary in large‐size anal tumors with the combination of neoadjuvant chemoradiation followed by wide excision of these.^1^ It is the responsibility of the treating physician, relying on independent experience and knowledge, to determine the best course of treatment for the patient.^2^

Early diagnosis of anal warts is critical to prevent progression of HSIL and improve patient outcomes.

## CONFLICT OF INTEREST

The author(s) declared no potential conflicts of interest with respect to the research, authorship, and/or publication of this article.

## AUTHOR CONTRIBUTION

FM and EI: contributed to the clinical data collection and prepared the case report; IM and PT: contributed to the design of the case report presentation and performed the final revision of the manuscript.
